# Neuron–glia crosstalk in health and disease: fractalkine and CX_3_CR1 take centre stage

**DOI:** 10.1098/rsob.130181

**Published:** 2013-12

**Authors:** Graham K. Sheridan, Keith J. Murphy

**Affiliations:** 1 Department of Physiology, Development and Neuroscience, University of Cambridge, Cambridge CB2 3DY, UK; 2 Neurotherapeutics Research Group, UCD School of Biomolecular and Biomedical Science, Conway Institute, University College Dublin, Belfield, Dublin 4, Ireland

**Keywords:** fractalkine, CX_3_CR1, synaptic plasticity, Alzheimer's disease, microglia, ischaemia

## Abstract

An essential aspect of normal brain function is the bidirectional interaction and communication between neurons and neighbouring glial cells. To this end, the brain has evolved ligand–receptor partnerships that facilitate crosstalk between different cell types. The chemokine, fractalkine (FKN), is expressed on neuronal cells, and its receptor, CX_3_CR1, is predominantly expressed on microglia. This review focuses on several important functional roles for FKN/CX_3_CR1 in both health and disease of the central nervous system. It has been posited that FKN is involved in microglial infiltration of the brain during development. Microglia, in turn, are implicated in the developmental synaptic pruning that occurs during brain maturation. The abundance of FKN on mature hippocampal neurons suggests a homeostatic non-inflammatory role in mechanisms of learning and memory. There is substantial evidence describing a role for FKN in hippocampal synaptic plasticity. FKN, on the one hand, appears to prevent excess microglial activation in the absence of injury while promoting activation of microglia and astrocytes during inflammatory episodes. Thus, FKN appears to be neuroprotective in some settings, whereas it contributes to neuronal damage in others. Many progressive neuroinflammatory disorders that are associated with increased microglial activation, such as Alzheimer's disease, show disruption of the FKN/CX_3_CR1 communication system. Thus, targeting CX_3_CR1 receptor hyperactivation with specific antagonists in such neuroinflammatory conditions may eventually lead to novel neurotherapeutics.

## 2. Introduction

In recent years, there has been an explosion in our understanding of how non-neuronal cells play crucial roles in many functions of the central nervous system (CNS). At present, these non-neuronal cell types are broadly categorized into (i) astrocytes, (ii) radial glia, (iii) oligodendrocytes, (iv) ependymal cells and (v) microglia. There exists further subcategories for each group and, more recently, NG2-positive glia are widely considered a distinct cell type [1–7]. The concept that the brain houses both neurons and ‘neuroglia’ was introduced in 1856 by the neuropathologist, Rudolf Virchow [8]. However, others that came after are credited with refining the classification of the various non-neuronal cell types present in the brain. Between them, Golgi [9], Andriezen [10] and Cajal [11] defined several distinct types of glial cells in the CNS. It was not until 1919, however, that Rio-Hortega, a student of Cajal, described microglia by labelling them with a modified silver carbonate stain [12]. Microglia are fundamentally distinct from other brain cells, being derived from primitive peripheral myeloid progenitors that arise during embryogenesis [13,14]. Microglia are the resident phagocytic cells in the brain, taking part in immune-mediated defence mechanisms and clearing damaged cell debris [15,16]. Previously, it was thought that microglia, in their resting state, are relatively quiescent. More recent work suggests that microglia are constantly active and surveying their surroundings [17,18]. Microglia are now implicated in synapse pruning, during both development and throughout adulthood, and therefore play a role in regulating homeostatic synaptic plasticity [19].

Together with astrocytes, microglia can release neuromodulatory chemicals that influence neuronal firing and intracellular signalling. When first described, astrocytes were seen merely as structural scaffolding to support and cushion neuronal cells within the brain, in essence, to fill the gaps between neurons [20]. However, that idea was quickly dismissed by Golgi who suggested that the function of astrocytes may be to provide nutrients for neurons. Cajal, however, did not fully agree with either of these explanations [8]. Recent evidence suggests that astrocytes serve as much more than a nutrient supply or supportive scaffolding to protect neural networks [21]. As mentioned, they release factors that modulate neurotransmission [22–24] and more recently have been suggested to possess their own repertoire of gliotransmitters [25–31]. The important roles played by glial cells in normal and pathological brain functioning are growing, and a more complete picture of neuron–glia interactions is beginning to emerge.

Glial cells are now accepted as key neuromodulators at every stage of development and adulthood and, therefore, must possess multiple mechanisms of communication with neuronal cell types. One method in which neurons and microglia are thought to communicate with one another is through neuronally expressed fractalkine (FKN; also known as CX_3_CL1 in the new chemokine nomenclature) [32]. FKN is expressed at the cell membrane of many neurons and binds to and activates CX_3_CR1 receptors on microglia [33]. Therefore, neuronally derived FKN can induce effects in microglia that may, in response, release neuromodulators that act back upon the same neuron and others in close proximity [34]. This neuron–glia crosstalk is the basis of this review, and we focus on the role played by FKN and CX_3_CR1 receptors in health and disease of the CNS.

## 3. Fractalkine: structure and cellular localization

Chemokines are abundantly expressed in many areas of the brain and spinal cord during development [35]. They regulate essential functions, including cell migration and differentiation [36]. Chemokines play diverse functions in the CNS during development, and later, throughout adulthood, they continue to mediate cell–cell communication [37] and regulate key functions such as neuroprotection following injury [33,38–45].

There are four distinct subfamilies of chemokines (α, β, γ and δ); categorized based on their amino acid structure. Most chemokines (except the γ subfamily) contain four conserved cysteine residues that form disulfide bonds to produce the defining chemokine domain. FKN is the only member of the δ subfamily of chemokines containing three amino acids (X_3_) separating the first two cysteine (C) residues [35]. It is also unusual in that it appears to bind only one receptor, the seven transmembrane G_i_ protein-coupled CX_3_CR1; many other chemokine members exhibit more promiscuous binding activity than FKN. The full-length molecule is larger than most other chemokines, containing approximately 373 amino acid residues compared with the more common 70–80 amino acid size range. FKN exists in two distinct forms. The first is an approximately 95 kDa full-length membrane-bound form that possesses a 76-amino acid N-terminal chemokine domain, a 241-amino acid glycosylated mucin-like stalk, an 18-amino acid hydrophobic transmembrane region and a 37-amino acid intracellular C-terminal domain. The second is an approximately 70 kDa soluble form that contains the N-terminal chemokine domain. The extracellular chemokine domain of FKN is proteolytically cleaved from the membrane-bound fraction by the lysosomal cysteine protease, cathepsin S and members of the ADAM (a disintegrin and metalloproteinase) family such as ADAM-10 and ADAM-17 (also known as TACE: tumour necrosis factor (TNF)-α-converting enzyme) [46–49]. The chemokine domain of FKN remains as a monomer in solution, as opposed to forming dimers which is more common for other chemokines [50]. The soluble chemokine domain of FKN, when cleaved, can act as a signalling molecule and can bind microglial-expressed CX_3_CR1 receptors [51], whereas its membrane-tethered mucin stalk can serve as a cell adhesion molecule [52] for microglia and infiltrating leucocytes during an inflammatory episode [53].

Unlike most chemokines, FKN is constitutively expressed in the CNS with particularly high levels in hippocampal neurons [34]. Astrocytes can also express FKN [33], although at relatively lower levels than neurons, whereas microglia appear not to express FKN mRNA transcripts. Hatori *et al*. [33] also report that neurons and microglia express CX_3_CR1 mRNA, whereas astrocytes do not. Therefore, neurons and astrocytes expressing FKN can signal to neurons and microglial cell types possessing CX_3_CR1 receptors. In this way, neurons may regulate microglial proliferation, because exogenous FKN can increase the number of BrdU-labelled microglia [33]. This may have implications in pathophysiological insults, including stroke, where FKN is upregulated and microglial numbers increase around the sites of neuronal damage [54]. Thus, cross-communication between FKN-expressing neurons and CX_3_CR1-containing microglia may potentially be an important factor in many CNS-related pathologies. It is also likely, however, that the sustained high levels of FKN in the brain throughout adulthood serve normal physiological functions in addition to a rapid response mechanism in times of traumatic injury.

## 4. Activators and repressors of fractalkine and CX_3_CR1 expression

Cleavage of the extreme N-terminal chemokine domain releases soluble FKN which can function as a signalling molecule and activate CX_3_CR1 receptors on neighbouring cells. FKN is constitutively expressed by certain neurons in the CNS but its expression levels can also be increased by several stimulators. For example, the treatment of rat aortic smooth muscle cells (SMCs) with the pro-inflammatory cytokine, TNF-α, induces the expression of FKN and CX_3_CR1 in a nuclear factor κB (NF-κB)-dependent manner [55]. Moreover, FKN itself can induce further FKN expression in a pertussis toxin (PTX)-sensitive and G protein-dependent manner. FKN autoregulation was shown to occur in this SMC type through a signalling cascade involving phosphoinositide 3-kinase (PI3K), phosphoinositide-dependent kinase 1 (PDK1), Akt, NIK, IKK and NF-κB activation [55]. Whether these same intracellular signalling cascades are recapitulated in CNS neurons following TNF-α exposure remains to be confirmed.

FKN has been shown to inhibit lipopolysaccharide (LPS)-induced TNF-α release from microglia [56], suggesting anti-inflammatory actions of FKN. Interestingly, when mixed neuron–glial cultures are prepared from CX_3_CR1 knockout (CX_3_CR1^−/−^) mice and stimulated with LPS, microglial cells in these mixed cultures release a reduced amount of TNF-α, nitric oxide (NO) and superoxide [57]. This suggests that the CX_3_CR1 receptor is involved in the release of pro-inflammatory substances from activated microglia. Therefore, by disrupting ‘normal’ FKN/CX_3_CR1 communication, it seems possible to switch the actions of FKN from anti-inflammatory to pro-inflammatory in nature.

By contrast, astrocytes do not constitutively express FKN protein. Astrocytes that are treated with certain pro-inflammatory cytokines such as TNF-α and IL-1β, however, upregulate expression of FKN in a time-dependent manner. Treatment of astrocytes with TNF-α induces FKN expression after 12 h and levels peak at 24 h. IL-1β, on the other hand, increased FKN expression much earlier after 2 h, peaking between 4 and 8 h post-stimulation [58]. It appears, therefore, that FKN may be upregulated in astrocytes in response to several pro-inflammatory signals, which can occur in conditions such as stroke, multiple sclerosis (MS) and Alzheimer's disease (AD). This increased expression of FKN could, in theory, modulate the release of further pro-inflammatory stimuli from microglial cell types, thus protecting susceptible neurons from neurotoxicity. FKN upregulation and release in such neuroinflammatory situations may, therefore, be neuroprotective and serve an overall anti-inflammatory action in the CNS. In later sections, however, we highlight some caveats when using FKN^−/−^ and CX_3_CR1^−/−^ mice to study whether FKN is neuroprotective or neurodamaging. The use of knockout animals that have had either FKN or CX_3_CR1 deleted from conception and allowed mature to adulthood with disruption to the FKN/CX_3_CR1 communication system can respond to FKN treatment differently to wild-type animals following inflammatory and neurodegenerative insults. Therefore, like many cytokines and chemokines studied in recent years, FKN can be either anti-inflammatory or neurodamaging depending on the context. The timing of administration (i.e. pre- or post-insult) and the concentration of FKN will be important factors in determining the response of neurons to potentially neurotoxic injury.

## 5. Fractalkine and CX_3_CR1-mediated intracellular signalling in neurons and microglia

**Figure 1.**
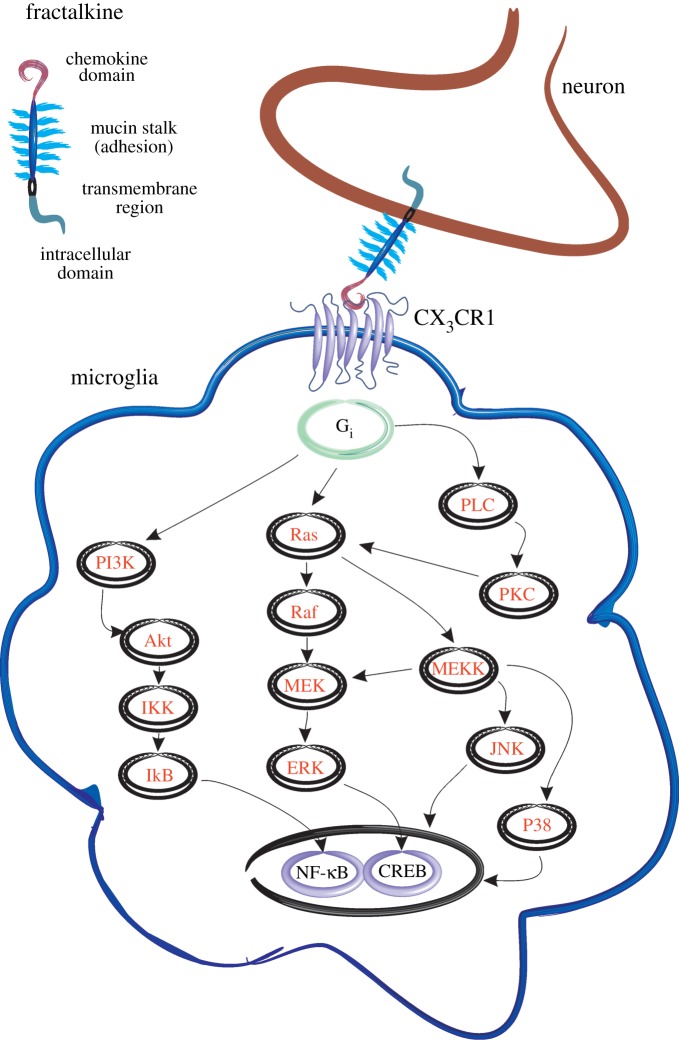
Fractalkine and CX_3_CR1 expression and signalling. Fractalkine is a large chemokine molecule consisting of four major functional regions. These include an N-terminal chemokine domain which can be cleaved by metalloproteinases such as ADAM10, TACE and the lysosomal cysteine protease, cathepsin S. The glycosylated mucin-like stalk is thought to be involved in cell adhesion, with particular affinity for migrating leucocytes at sites of inflammation. Fractalkine also contains a hydrophobic transmembrane region and intracellular C-terminal domain. Neuronally expressed membrane-bound and soluble cleaved fractalkine can bind to its receptor, CX_3_CR1, which is G protein-coupled and transduces several well-characterized signalling pathways leading to activation of transcription factors, including NF-κB and CREB. FKN, fractalkine; Gi, heterotrimeric G protein-coupled to G_i_ protein; PI3K, phosphatidylinositide 3-kinases; Ras, small GTPase; Raf, small GTPase; PLC, phospholipase C; PKC, protein kinase C; Akt, serine/threonine-specific protein kinase; MEK, mitogen-activated protein kinase kinase; MEKK, MAP3kinase; p38, p38 mitogen-activated protein kinase; IkB, inhibitor of kappa B; IKK, inhibitor of kappa B kinase; ERK, extracellular signal-regulated kinases; JNK, c-Jun N-terminal kinase; NFκB, nuclear factor kappa B; CREB, cAMP response element-binding protein.

The FKN receptor, CX_3_CR1, is reportedly expressed on microglia and on neurons [33,39,45,58–64]. CX_3_CR1 is a seven transmembrane domain receptor coupled to G_i_ and G_z_ subtypes of G proteins [65], activation of which is linked to several intracellular second messengers (figure 1) [55]. In microglia, FKN has been shown to decrease LPS-induced MHCII and CD40 mRNA levels *in vitro.* Moreover, microglial IL-1β protein expression is also attenuated in cells treated with FKN and these anti-inflammatory effects are Akt (also known as PKB) and PI3-kinase-dependent [66]. FKN also rapidly increases Akt activation in microglia in a dose- and time-dependent manner, as measured by phosphorylation of histone 2B. Astrocytes, on the other hand, do not demonstrate this intracellular signalling cascade post-FKN exposure [58].

Several studies have also used *in vitro* cell culture systems to investigate the effects of FKN on neuronal cell types. Treatment of human neurons with FKN induced transient phosphorylation of ERK1/2 within 1 min and Akt within 10 min of exposure [43]. The same authors also showed that FKN can significantly inhibit NMDA-induced calcium influx in neurons, and this effect is insensitive to PTX pre-treatment. This attenuation of calcium influx is, however, abolished by inhibition of ERK1/2 signalling. FKN also inhibits NMDA-mediated apoptosis and this neuroprotective effect is abolished by blocking Akt and ERK1/2 signalling pathways [43]. This work demonstrates that FKN can have direct effects on neurons and that these actions may promote neuroprotection following potentially toxic insults.

It is important to differentiate between FKN's direct effects on neuronal cells versus glial cell-mediated neuromodulatory effects. When hippocampal neurons are cultured with a glial ‘feeder layer’, a pure neuronal cell population can be achieved. Treatment of a pure neuronal hippocampal culture with soluble FKN activates ERK1/2, whereas no JNK or p38 MAPK upregulation occurs. FKN also activates the transcription factor, CREB, in hippocampal neurons [39]. Removal of this glial feeder layer causes a 75% decrease in basal Akt phosphorylation in neurons. Subsequent treatment of these neurons with FKN induces a significant increase in phospho-Akt levels which is PI3-kinase-dependent [59]. Inhibiting PI3-kinase activity in neurons abolishes FKN-mediated neuroprotection. FKN also induces translocation of the p65 subunit of NF-κB to the nucleus in hippocampal neurons and this is prevented by a specific inhibitor of PI3-kinase, suggesting that FKN activates NF-κB through Akt [59]. These results give credence to a possible role of FKN in modulating neuronal synaptic plasticity as the transcription factors CREB and NF-κB are intimately linked to complex temporal gene regulation required for learning and memory [67].

## 6. Fractalkine and CX_3_CR1 in CNS development

During early postnatal development, neurons forge many more synaptic connections than is necessary for normal adult brain function. Consequently, a portion of these connections is not maintained into adulthood but removed through a process of activity-dependent pruning [68]. More recently, microglial cells have been suggested to play a key role in developmental synaptic pruning. Paolicelli *et al*. [69] report that microglia actively phagocytose synapses during the first few weeks of mouse brain maturation. Using CX_3_CR1^GFP/GFP^ mice, in which microglia are fluorescently labelled (with green fluorescent protein, GFP) and the FKN receptor knocked out (CX_3_CR1^−/−^), they quantified the number of PSD-95-labelled dendritic spines on CA1 neurons during the first five weeks of postnatal development. They found that during the second–third weeks, CX_3_CR1^−/−^ mice possess more synapses than wild-type mice. Interestingly, CX_3_CR1^−/−^ mice also express reduced numbers of microglia in the CA1 region of the hippocampus during weeks 2–4 of postnatal development. The authors suggest that FKN signalling during development may act as a chemotropic agent to attract microglial cells into the brain. Therefore, knocking out the CX_3_CR1 receptor would render microglia blind to the attractive FKN cue and lead to reduced numbers of microglia in the brain of CX_3_CR1^−/−^ mice. If microglia are actively involved in synapse pruning at this developmental stage, then a reduced number of microglial cells could explain the higher density of synapses in the brains of CX_3_CR1^−/−^ mice [69]. However, an alternative explanation might be that FKN/CX_3_CR1 signalling is required for proper microglial-recognition of synaptic boutons before and/or during engulfment and phagocytosis.

In contrast to the action on microglia, astrocytes show little or no cell migratory response to FKN. Even activation of astrocytes with TNF-α or IL-1β does not induce a migratory phenotype in response to FKN exposure. Moreover, Lauro *et al*. [70] showed that neurons from the hippocampus and cerebellum display reduced cell migration in *in vitro* assays in response to FKN. This inhibitory effect of FKN on neuronal cell migration was dependent on activation of the CX_3_CR1 receptor, was PTX-sensitive and dependent on PI3-kinase activity. The authors suggest that FKN may inhibit neuronal cell migration by increasing binding and adhesion to laminin, a component of the extracellular matrix [70]. Interestingly, FKN has the opposite effect on microglial cells and decreases their adhesion to poly-l-lysine-coated surfaces in *in vitro* assays. Blocking the CX_3_CR1 receptor and inhibiting G_i_ protein-coupled responses with PTX diminishes microglial migration in response to FKN [58]. This supports the hypothesis that FKN may act as an attractive microglial guidance cue during development *in vivo* and that FKN/CX_3_CR1 signalling promotes the population of CNS tissue with peripherally derived cell types.

In the developing barrel field of the somatosensory cortex, FKN/CX_3_CR1 signalling is also thought to regulate microglial influx, and population of sites where developing thalamocortical synapses are concentrated (i.e. barrel centres). Hoshiko *et al*. [71] showed that microglial cell infiltration into the barrel centres occurs around postnatal day 5 (P5) in the mouse; at a time when FKN is abundantly expressed in these regions. In CX_3_CR1^GFP/GFP^ receptor knockout mice, microglial entry into the barrel centres is delayed by a few days, but is indistinguishable from wild-type mice at postnatal day 9 (P9). The absence of CX_3_CR1 also delays the maturation of functional glutamate receptors evidenced by the fact that the AMPAR/NMDAR ratio at P9 was significantly lower in CX_3_CR1^−/−^ than in CX_3_CR1^+/−^ mice. Moreover, the developmental switch from GluN2B to GluN2A-containing NMDA receptors that is known to occur between the first and second postnatal weeks in the thalamocortical area of the mouse was delayed in CX_3_CR1^−/−^ mice but this delay was only transient [71]. This lends support to the notion that microglia influence synaptic maturation during development and that FKN signalling contributes to this process.

In the adult brain, neuronally derived FKN is thought to maintain microglia in a quiescent state. Lyons *et al*. [66] have shown that in aged rats, the levels of FKN in the hippocampus decrease and this correlates with an increase in CX_3_CR1 expression and microglial cell activation as evidenced by increased MHCII, CD40 mRNA and IL-1β protein levels. Thus, FKN may play a role in the homeostatic suppression of microglial activation. The seemingly natural decline in neuronally expressed FKN in the hippocampus of aged animals [66] might contribute to increased microglial activation. If the same occurs in humans, this decrease in FKN expression over time could contribute to several cognitive and neurodegenerative disorders that are more common in the elderly.

## 7. Fractalkine and CX_3_CR1 modulate synaptic plasticity

FKN mRNA and protein levels are abundant in the uninjured adult hippocampus, an observation that prompted some to investigate if FKN plays a physiological role in learning and memory formation. Many recent publications suggest a functional role for both FKN and CX_3_CR1 in the regulation of neurotransmission and synaptic plasticity [72–76]. There still exists some controversy as to whether CX_3_CR1 receptors are present on neurons *in vivo*; even though several papers have reported that CX_3_CR1 does, indeed, reside on neurons, particularly hippocampal neurons [45,59]. Meucci *et al*. [39] reported that FKN induces calcium influx in pure hippocampal neuronal culture *in vitro* in the absence of any microglial contamination. Heinisch & Kirby [64] also have shown that CX_3_CR1 localizes to the perinuclear region of serotonergic (5-HT) neurons in the raphe *in vivo* in addition to exhibiting microglial expression.

As discussed above, there is mounting evidence that FKN exerts direct actions on neurons through the CX_3_CR1 receptor. For instance, FKN reduces spontaneous glutamate release and post-synaptic glutamate currents [39,45]; the latter being linked to dephosphorylation of the GluR1 AMPA receptor subunit [73]. FKN also decreases the frequency, but not the amplitude, of spontaneous mini excitatory post-synaptic currents (mEPSCs) from hippocampal neurons in culture [39]. Several lines of evidence indicate that the predominantly inhibitory action of FKN in the hippocampus may underlie an important role in synaptic scaling, and homeostasis of the hippocampal network that is necessary for memory-associated synaptic plasticity [72,74,77]. For example, FKN causes a reversible decrease in field excitatory post-synaptic potentials (fEPSPs) in the CA1 region of mouse hippocampal slices. This depression is rapid in onset and dose-dependent but reversible, as the fEPSP amplitude recovers within 30 min of FKN washout [72]. This depression of fEPSPs is absent in CX_3_CR1^−/−^ mice. Similarly, FKN causes a significant reduction in EPSCs from stimulated CA1 pyramidal neurons [73]. The ability of FKN to depress EPSCs appears to be post-synaptically mediated, because paired-pulse facilitation (PPF) is unaltered following FKN treatment. Moreover, EPSC depression is absent in CX_3_CR1^−/−^ mice. FKN's depressive actions on EPSCs are dependent on calcium entry into the cell but independent of NMDA receptor activation [73]. FKN inhibits forskolin-induced Ser845 phosphorylation of the GluR1 subunit of the AMPA receptor which contributes to inhibition of EPSC amplitude. Interestingly, FKN does not induce EPSC depression when the baseline synaptic stimulation protocol was suspended until after FKN washout, indicating that FKN-mediated depression is activity-dependent. FKN reduces AMPA receptor currents and the larger the current amplitude, the greater is the depression by FKN [73]. Taken together, these pieces of evidence suggest that FKN modulates AMPA receptors at active glutamatergic synapses and depresses synaptic transmission. Interestingly, prior induction of long-term depression abolishes the inhibitory effect of FKN on synaptic transmission arguing that these forms of synaptic depression may overlap at the molecular level [72].

Interestingly, there seems to be an important temporal component to the inhibitory actions of FKN on long-term potentiation (LTP). FKN, when present in the circulating perfusate prior to high-frequency stimulation (HFS), inhibits LTP in the CA1 region of acute hippocampal slices. When added just a few minutes after LTP induction, however, FKN has no dampening effect on the maintenance phase of LTP [78]. This inhibitory action of FKN on LTP is mediated through the CX_3_CR1 receptor because it was absent in CX_3_CR1^−/−^ mice.

There is also evidence to suggest that adenosine and adenosine receptors are modulated by FKN activity in the hippocampus. FKN has been shown to cause the release of adenosine from microglia [79]. Exposure of acute hippocampal slices to a broad spectrum adenosine receptor antagonist abolished the inhibitory effect of FKN on LTP. Maggi *et al*. [78] pinpointed the A_3_R adenosine receptor as the crucial subtype mediating inhibition of LTP by FKN in the CA1. This is in contrast to FKN-mediated neuroprotection which appears to be dependent on adenosine-1 receptor (A_1_R) activation [79]. In agreement with this study, the same group showed that FKN does not induce EPSC depression in A_3_R^−/−^ mice, but that this feature is present in both A_1_R^−/−^ and/or A_2A_R^−/−^ mice [74]. More recently, they also showed that FKN potentiates the NMDA receptor component of the fEPSP in the hippocampal CA1 region [80]. The authors propose that FKN activates CX_3_CR1 receptors on microglia which induces adenosine release. This adenosine may, in turn, activate A_2A_R receptors on microglia (and possibly astrocytes) causing the release of d-serine which acts as a co-agonist at the NMDA receptor, thus potentiating NMDA-mediated fEPSPs. Interestingly, this might explain why FKN inhibits LTP when administered prior to LTP induction but not if added just a few minutes post-HFS. The increase in intracellular calcium that accompanies NMDA fEPSP potentiation by FKN may disrupt LTP induction which has been shown to depend on the duration and amplitude of Ca^2+^ elevations as well as the metaplastic activation state of the NMDA receptors involved [81,82].

CX_3_CR1^−/−^ and CX_3_CR1^+/−^ mice display deficits in motor learning as assessed by standard rotarod training techniques. There is, however, no difference between CX_3_CR1^−/−^, CX_3_CR1^+/−^ and wild-type mice in spontaneous locomotor activity as assessed by the open field test. Similarly, CX_3_CR1^−/−^ and CX_3_CR1^+/−^ mice were no more anxious than control mice as assessed by the elevated plus maze [76]. Using standard fear-conditioning paradigms, CX_3_CR1^−/−^ and CX_3_CR1^+/−^ mice were assessed for deficits in associative learning and memory. During the training period, CX_3_CR1^−/−^ and CX_3_CR1^+/−^ mice displayed similar freezing behaviour to wild-type mice. When the mice were placed back into the chamber 24 h later, however, CX_3_CR1^−/−^ and CX_3_CR1^+/−^ mice displayed a reduction in their freezing behaviour. This ‘context-specific’ type of associative memory is thought to be predominantly hippocampal-dependent. Interestingly, when the CX_3_CR1^−/−^ and CX_3_CR1^+/−^ mice were placed into a novel environment and exposed to the conditioning stimulus, the animals froze for the same amount of time as their wild-type counterparts [76]. This ‘conditioning-specific’ type of associative memory is thought to depend on both the hippocampus and amygdala suggesting that CX_3_CR1^−/−^ and CX_3_CR1^+/−^ mice may display hippocampal-specific deficits in cognition. In agreement with this notion is the fact that CX_3_CR1^−/−^ and CX_3_CR1^+/−^ mice perform worse in probe trials of the hidden water maze task—a hippocampal-dependent memory paradigm. Rogers *et al*. [76] also looked at measures of synaptic plasticity in CX_3_CR1^−/−^ and CX_3_CR1^+/−^ mice. Basal synaptic transmission as well as PPF in the hippocampus was normal. However, CX_3_CR1^−/−^ and CX_3_CR1^+/−^ mice showed a reduction in LTP in the CA1 stratum radiatum. This may be explained by the higher basal levels of IL-1β in the hippocampus of CX_3_CR1^−/−^ and CX3CR1^+/−^ mice compared with wild-type. The origin of this excess IL-1β may be microglial cells, because CX_3_CR1^−/−^ mice possess more activated microglia in the hippocampus. Treating hippocampal slices with IL-1 receptor antagonist (IL-1RA) rescued the deficits in LTP seen in CX_3_CR1^−/−^ mice. Moreover, treating CX_3_CR1^−/−^ mice with IL-1RA reverses the deficits in learning and memory seen in contextual fear-conditioning and water maze spatial memory training. TNF-α levels were also elevated in the cerebellum of CX_3_CR1^−/−^ mice suggesting an overall increase in inflammatory-associated protein levels in the brains of CX_3_CR1^−/−^ and CX_3_CR1^+/−^ mice [76].

**Figure 2.**
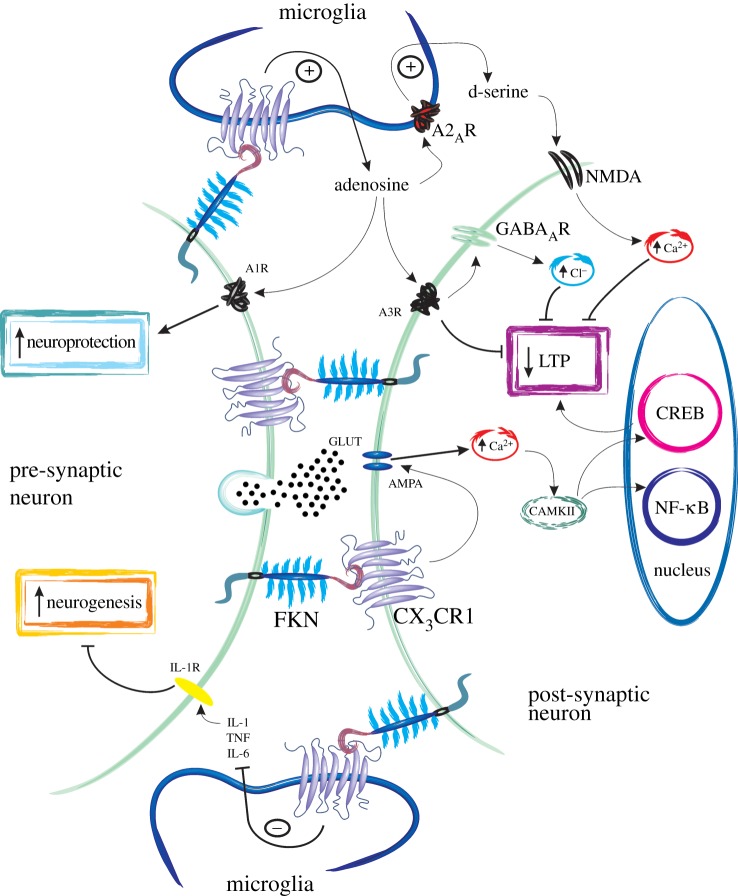
Fractalkine and CX_3_CR1 in synaptic plasticity, neurogenesis and neuroprotection. Schematic diagram describing several mechanisms of action of fractalkine (FKN) in modulating neuronal function. Hippocampal neurons, in particular, express high levels of FKN and CX_3_CR1 receptors. Microglia also possess CX_3_CR1 and can release several chemicals that modulate neurotransmission and synaptic plasticity. First, FKN acting through CX_3_CR1 modulates AMPA receptor phosphorylation leading to increased calcium (Ca^2+^) entry and inhibition of both excitatory post-synaptic potentials (EPSPs) and long-term potentiation (LTP). FKN can also increase inhibitory post-synaptic currents (IPSCs), possibly by enhancing neuronal responsiveness to GABA-mediated chloride entry. How FKN enhances IPSCs remains unknown, but this may be due to FKN activating CX_3_CR1 on microglia and causing the release of adenosine. This, in turn, could activate A_3_R receptors on neurons, kick-starting a signalling cascade which results in modulation of GABA_A_ receptors to increase their sensitivity to GABA. Adenosine may also activate A_2A_R on microglial cells and induce the release of d-serine which acts as a co-agonist at the NMDA receptor leading to increased calcium entry. In this way, FKN may also inhibit LTP induction and modulate synaptic plasticity. The adenosine released by microglia has also been suggested to play a role in neuroprotection by activating A_1_R receptor subtypes on neurons. Finally, FKN may play a role in hippocampal neurogenesis by inhibiting the release of IL-1β from microglial cell types. Much of this schematic diagram is speculative and based on our limited current knowledge of the interplay between FKN and CX_3_CR1 in CNS neurotransmission. There is still much work to be done to dissect the signalling cascades involved in FKN-mediated neuromodulation.

Taken together, there is substantial evidence that FKN and CX_3_CR1 signalling play several crucial roles in synaptic plasticity, learning and memory and FKN likely contributes to maintaining proper homeostasis of synaptic transmission in the hippocampus (figure 2).

## 8. Fractalkine and CX_3_CR1 role in neurogenesis

Hippocampal neurogenesis is often correlated with cognitive function [83,84] and new-born dentate granule cells are thought to be involved in spatial memory formation [85,86]. Both CX_3_CR1^−/−^ and CX_3_CR1^+/−^ mice display reduced hippocampal neurogenesis compared with wild-type controls [76,87]. Moreover, blocking CX_3_CR1 activity in young adult rats (three months old) results in an attenuation of neurogenesis. Given the role of hippocampal neurogenesis in learning and memory, reduced neurogenic rate may underlie impairments observed in CX_3_CR1^−/−^ mice in hippocampal-dependent memory tasks.

It is widely accepted that cognitive ability decreases with age and this decline correlates with reduced levels of hippocampal neurogenesis. Interestingly, treatment of older animals with exogenous FKN reverses this age-related decrease in hippocampal neurogenesis [87]. In support of the observation that FKN limits pro-inflammatory cytokine release in the hippocampus, Bachstetter *et al*. [87] showed that administering an IL-1 receptor antagonist (IL-1RA) attenuated the decrease in hippocampal neurogenesis which occurs as a result of blocking CX_3_CR1 receptors. This suggests that FKN may activate CX_3_CR1 on microglial cells to attenuate IL-1β release to maintain the rate of hippocampal neurogenesis.

## 9. Fractalkine and CX_3_CR1 mediate neuroprotection

As well as serving to inhibit over-activation of the hippocampal network under physiological conditions, FKN may also act as a first-line defence response to neuronal injury and neuroinflammation. Pathological levels of glutamate and excitotoxicity can lead to a TACE-mediated increase in soluble FKN, thus limiting neuronal damage [45,51,73,88,89]. As well as attenuating glutamate-mediated excitation, FKN can enhance the responsiveness of at least some neurons to GABA inhibition. Specifically, FKN inhibits serotonin neurons of the raphe by enhancing the activity of GABAergic receptors [64]. In fact, over 70% of raphe serotonin neurons exhibit FKN/CX_3_CR1 co-localization further suggesting that FKN has direct actions on neurons as opposed to indirect actions through microglial-mediated neuromodulation. If the direct actions of FKN on GABA-sensitivity are exclusive to serotonin-containing neurons in the dorsal raphe nucleus, then this mechanism presents a novel drug target for mood-related disorders such as anxiety and depression. However, a recent study by Roseti *et al*. [90] suggests that FKN may also modulate GABA_A_ receptor-mediated currents in other brain regions, such as the hippocampus and cortex, and that this action has relevance for disorders such as mesial temporal lobe epilepsy.

## 10. Fractalkine and CX_3_CR1 control microglia-mediated neurotoxicity

Microglial cells are the resident macrophages of the brain and are activated in response to neuronal injury [91]. Cardona *et al.* [92] made use of the CX_3_CR1^GFP/GFP^ receptor knockout mouse to study the role of FKN and CX_3_CR1 signalling in several models of microglial-induced neurotoxicity. CX_3_CR1^−/−^ mice demonstrated significantly greater microglial activation in response to intraperitoneal (i.p.) administration of LPS. As only microglial cells are labelled with GFP in these CX_3_CR1^−/−^ mice, Cardona *et al*. developed an adoptive transfer technique that allowed them inject LPS-activated microglial cells, deficient in CX_3_CR1 receptors, into the brains of wild-type animals. The ‘behaviours’ of these GFP-labelled microglial cells were compared with those taken from CX_3_CR1^+/−^ mice. Interestingly, CX_3_CR1^+/−^ microglial cells migrated far from the site of injection and seemed to preferentially invade white matter tracts of the CNS parenchyma. CX_3_CR1^−/−^ microglia, however, localized to the site of injection in the frontal cortex of wild-type mice. Moreover, there was significantly more neuronal cell loss surrounding activated GFP+ microglia transplanted from CX_3_CR1^−/−^ mice than in wild-type brains that received CX_3_CR1^+/−^ microglial cells. The authors attributed this increased neuronal cell death to an increased production of IL-1β (but not TNF-α, lymphotoxin or IL-6) from CX_3_CR1^−/−^ microglia upon LPS stimulation. Indeed, when IL-1RA (IL-1 receptor antagonist) was added along with the adoptive transfer microglia, neuronal cell death was reduced significantly and IL-1RA seemed to restore the migratory activity of CX_3_CR1^−/−^ cells from the site of injection.

Cardona *et al*. [92] also evaluated the role of FKN/CX_3_CR1 signalling in the 1-methyl-4-phenyl-1,2,3,6-tetra-hydropyridine (MPTP)-induced neurodegenerative mouse model of Parkinson's disease (PD). Dopaminergic cell death in the substantia nigra pars compacta of MPTP-injected CX_3_CR1^−/−^ mice was significantly more substantial than that seen in wild-type mice. Moreover, FKN knockout (CX_3_CL1^−/−^) mice displayed similar enhancement of neuronal loss to CX_3_CR1^−/−^ mice indicating that it is the perturbation of FKN-mediated modulation of microglial activity that worsens neuronal cell death, i.e. neuron–microglia crosstalk, rather than a specific loss of either FKN or its receptor. Pabon *et al*. [89] showed that FKN was neuroprotective in the 6-hydroxydopamine (6-OHDA) toxin-induced rat model of PD. In this model, 6-OHDA is infused in the striatum of rats causing selective neurotoxicity of dopaminergic neurons, thus mimicking the human form of PD. Microglial activation is thought to contribute to neurodegeneration which follows 6-OHDA administration. FKN infused into the rat striatum over the course of several weeks caused a significant reduction in microglial activation which correlated with marked neuroprotection of dopaminergic neurons [89].

Activation of microglial cells with LPS can convert the phenotype of microglial cells from a resting state into a phagocytic and neurotoxic form. Zujovic *et al*. [56] showed that microglia that were pre-exposed to LPS *in vitro* and subsequently added to a hippocampal neuronal culture were neurotoxic and caused 20% neuronal cell death. LPS induced the release of TNF-α from microglia which FKN treatment partially blocked. The addition of a neutralizing antibody against endogenous FKN enhanced neuronal cell death in this co-culture of LPS-activated microglia. These results suggest that tonic activation of CX_3_CR1 by endogenous FKN may serve as an anti-inflammatory signal that maintains microglial cells in a quiescent state. Mizuno *et al*. [93] showed that microglia which are activated by both LPS and interferon-gamma (IFN-γ) release NO, interleukin (IL)-6 and TNF-α. FKN dose-dependently inhibits neuronal cell death induced by activated microglia suggesting an intrinsic role for the high levels of FKN expressed on neurons in the adult CNS [93].

## 11. Fractalkine and CX_3_CR1 in ischaemic brain injury

Despite the evidence of a neuroprotective role for FKN presented above, there are data suggesting this chemokine is detrimental in some settings. Soriano *et al.* [94] generated mice that were deficient in FKN (CX_3_CL1^−/−^) in order to determine the role of FKN in ischaemic brain injury. They induced a transient focal cerebral ischaemia (2 h) and allowed a 22 h reperfusion of the middle cerebral artery before sacrificing the mice for histological analysis of tissue injury. Twenty-four per cent of the wild-type mice died in this 22 h period, whereas none of the 20 CX_3_CL1^−/−^ mice died. Moreover, there was a 28% reduction in the size of the ischaemic infarct in CX_3_CL1^−/−^ mice correlating well with the reduced mortality rate. This study suggests that FKN expression is detrimental to recovery post-ischaemic injury [94]. Cipriani *et al*. [95] performed a similar study on CX_3_CL1^−/−^ mice and also CX_3_CR1^−/−^ mice. They also found that CX_3_CL1^−/−^ mice possessed a smaller ischaemic infarction following middle cerebral arterial occlusion (MCAO) and interestingly, so did CX_3_CR1^−/−^ mice. Counterintuitively, Cipriani *et al*. also showed that administration of exogenous FKN slightly before MCAO to wild-type mice reduced the volume of the subsequent ischaemic infarct. Therefore, FKN is neuroprotective in wild-type mice that receive an ischaemic insult. Exogenous FKN administration had no effect in CX_3_CR1^−/−^ mice but did, however, increase the size of the infarct seen in CX_3_CL1^−/−^ mice. Therefore, mice that develop to adulthood with normal levels of FKN present in the brain (i.e. wild-types), respond favourably to FKN pre-treatment post-ischaemia. The story is further complicated, because exogenous FKN treatment is only beneficial at a reasonably narrow concentration range of between 15 and 70 pM; greater than 150 pM FKN becomes harmful in wild-types post-ischaemia. Interestingly, the neuroprotective effects of exogenously applied FKN were lost in A1R^−/−^ knockout mice [95].

**Figure 3.**
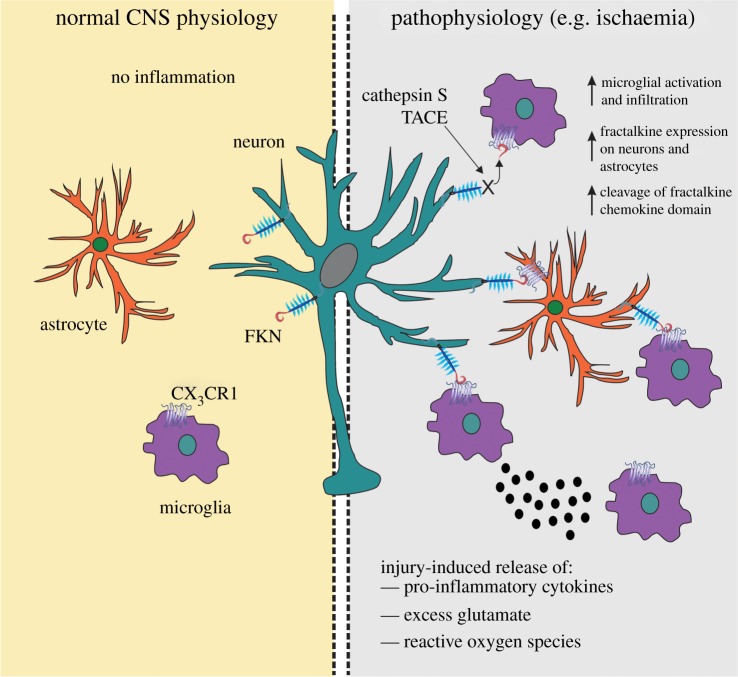
Fractalkine and CX3CR1 in neuroinflammatory conditions. In the uninjured brain under normal physiological conditions, fractalkine (FKN) is largely expressed on neurons and CX_3_CR1 receptors on microglial cells. FKN sequesters microglia in a quiescent ‘inactive’ state. Astrocytes are relatively devoid of FKN and CX_3_CR1 protein expression. Under a pathological insult, such as occurs following ischaemia, FKN can be upregulated on neuronal cells. FKN can also be cleaved by the metalloproteinase, TNF*α*-converting enzyme (TACE), and lysosomal cysteine protease, cathepsin S, released during injury. Upregulated levels of FKN can attract microglia to the site of inflammation, where they become activated and release pro-inflammatory mediators such as cytokines, reactive oxygen species (ROS) and glutamate. Astrocytes can also express FKN following an inflammatory insult and thus can communicate with both neurons and microglia via CX_3_CR1. The increased expression of FKN should have a net anti-inflammatory action and serve to limit inflammation in favour of functional recovery of CNS tissue.

The fact that a developmentally disrupted neuron–microglia communication system can alter whether FKN is protective or detrimental in ischaemic injury suggests that FKN/CX_3_CR1 signalling in microglia is important for their proper functioning. Under *in vitro* conditions that mimic an *in vivo* MCAO injury, i.e. oxygen–glucose deprivation, FKN caused a reduction in TNF-α release from microglia that were cultured from CX_3_CL1^−/−^ mice. Recently, a neuroprotective role for TNF-α in ischaemic brain injury has been described [96] and this may explain why exogenous FKN exacerbates the infarct volume in CX_3_CL1^−/−^ mice. The release of various factors from microglia at the site of injury can have both positive and negative effects on neuronal survival and normal microglia functioning appears disrupted in CX_3_CL1^−/−^ mice, because FKN does not alter TNF-α release from wild-type microglia. Dénes *et al*. [97] suggest that CX_3_CR1 is crucial for maintaining proper microglial functioning in the CNS. They also showed that the infarct size post-MCAO was significantly reduced in CX_3_CR1^−/−^ mice compared with wild-types and heterozygotes. Interestingly, they noted increased IL-1β expression in CX_3_CR1^+/−^ mice compared with knockouts, but the IL-1β was not associated with microglial cell types. Instead, astrocytes in CX_3_CR1^+/−^ mice produced elevated levels of IL-1β post-MCAO compared with knockout animals. This suggests that astrocytes, under certain conditions, may also express CX_3_CR1 receptors (figure 3) or, alternatively, that microglia lacking the CX_3_CR1 receptor assume a phenotype that can alter astrocytic function in times of stress such as an ischaemic event.

A more recent study by Pimentel-Coelho *et al*. [98] looked at sex-specific effects of FKN and CX_3_CR1 in a neonatal model of ischaemic-hypoxic injury. Three-day-old (P3) mice (male and female) were subjected to MCAO followed by a 40 min 8% oxygen challenge. They found that FKN mRNA levels are reduced in the CA3 and CA1 of the hippocampus, in both males and females, 24 h and 3 days post-ischaemia–hypoxia and this reduction in FKN mRNA was evident up to five weeks post-surgery. Twelve weeks post-ischaemia–hypoxia, wild-type and CX3CR1^−/−^ mice were tested for spatial learning ability and cognitive functioning in the T-water maze task. CX3CR1^−/−^ males subjected to neonatal ischaemia–hypoxia showed similar test scores to sham-operated CX3CR1^−/−^ males. Sham-operated CX3CR1^−/−^ females, however, performed better than their ischaemia–hypoxia-subjected counterparts [98]. This can be explained by the reduced levels of neuronal injury in wild-type females compared with CX3CR1^−/−^ females post-neonatal ischaemia–hypoxia. Therefore, CX3CR1 signalling seems to play a greater neuroprotective role in female compared with male mice following a neonatal ischaemic-hypoxic event.

## 12. Fractalkine and CX_3_CR1 in multiple sclerosis

The FKN/CX_3_CR1 signalling partnership may have a role to play in neuroinflammatory and autoimmune diseases of the CNS. MS is perhaps the quintessential autoimmune disorder of the CNS, characterized by inflammation and demyelinating lesions in the spinal cord and brain of affected patients [99,100]. The animal model that is regarded to most closely model the human disease is experimental autoimmune encephalomyelitis (EAE) [101,102]. Expression levels of FKN and CX_3_CR1 receptor have been found to change in and around the demyelinating lesions that accompany EAE and disease progression. Myelin oligodendrocyte glycoprotein (MOG)-induced EAE in rats causes a marked accumulation of CX_3_CR1-expressing microglia within brain lesions and sites of inflammation [103]. Notably, CX_3_CR1 mRNA increased in the periplaque regions of the CNS as well in early-active, late-active and also inactive demyelinated lesions, indicating the infiltration of microglia into these affected areas. Neuronal FKN levels in the same rats remained at control levels, but there was an increase in astrocyte-associated FKN expression at sites of inflammation. This is particularly interesting as it suggests that activated astrocytes may be involved in attracting microglia to the sites of inflammation through the upregulation of FKN on their surface membranes [103]. Neuronal FKN mRNA expression in the brains of rats induced with EAE is unaltered [104]. However, immuno-deficient α-myelin basic protein T-cell receptor transgenic mice that develop EAE spontaneously display upregulated FKN in brain microglia. Similarly, mice actively immunized with proteolytic peptide show increased expression of FKN in brain microglia [105]. In this regard, autoimmune dysfunction in the CNS may be a trigger for microglia themselves to upregulate FKN expression. This may contribute to disease progression, or more likely, be a mechanism by which microglia attempt to autoregulate their over-activation and return neighbouring microglia to a quiescent state.

In a recent study by Garcia *et al*. [106], the authors found that the disruption in FKN signalling that occurs on peripheral bone-marrow-derived cells in CX_3_CR1^−/−^ mice contributes to more severe EAE in these animals. CX_3_CR1^−/−^ mice immunized with MOG_35–55_ peptide displayed earlier onset and more severe EAE symptoms than wild-type animals. The same was true when EAE was induced by adoptive transfer of MOG_35–55_ reactive T cells. CX_3_CR1^−/−^ mice induced with EAE showed an accumulation of CD115^+^Ly6C^–^CD11c^+^ dendritic cells in the brain and this correlated with more pronounced demyelination as well as enhanced neuronal damage. CX_3_CR1^−/−^ mice suffering from EAE also showed over-expression of pro-inflammatory cytokines in CNS tissues. TNF-α levels in EAE-affected CX_3_CR1^−/−^ mice were higher than their diseased wild-type counterparts. Likewise, IFN-γ mRNA was higher in the cerebellum and spinal cord and IL-17 was more abundant in forebrain and cerebellar regions of CX_3_CR1^−/−^ mice induced with EAE. By contrast, levels of the anti-inflammatory cytokine, IL-10, was significantly higher in the spinal cord of EAE-wild-type mice compared with diseased CX_3_CR1^−/−^ animals [106]. Taken together, these results highlight the importance of proper FKN/CX_3_CR1 signalling in regulation of autoimmune responses and the possible role of CX_3_CR1 in MS in the human population. In particular, polymorphic variants of the CX_3_CR1 receptor which can affect FKN binding affinity as well as receptor expression have been identified. A study by Stojković *et al*. [107] suggests that there is a lower incidence of the CX3CR1^I249/T280^ haplotype in patients that display secondary-progressive MS compared with patients in the relapsing–remitting phase of the disease. This genetic study reveals a possible protective effect of the reference I249 allele on secondary-progressive MS when linked with the T280 allele [107].

## 13. Fractalkine and CX_3_CR1 in spinal cord injury

Spinal cord injury (SCI) induces a devastating trauma to neuronal cells and results in the destruction and severing of axons, leading to widespread neurodegeneration and inflammation in and around the site of injury [108]. After some time, microglial cells and monocyte-derived macrophages (MDMs) are recruited to the affected regions of the spinal cord [109]. Their role is presumably to promote functional recovery of damaged nervous tissue. They may, however, promote the formation of a glial scar which, in actuality, serves to inhibit functional recovery of damaged neuronal connections in favour of speeding up the healing process and increasing the chance of survival of the whole organism [110]. Donnelly *et al*. [111] have shown that CX_3_CR1^−/−^ mice, lacking the receptor for FKN, display a distinct repertoire of MDMs that infiltrate the injured spinal cord compared with wild-type mice. CX_3_CR1^−/−^ mice expressed relatively greater numbers of CCR2^+^ CNS macrophages post-SCI than wild-type mice, whereas wild-type animals displayed more iNOS^+^ MDMs in the injured spinal cord. CX_3_CR1^−/−^ microglia also produced less IL-6 and iNOS mRNA post-SCI. Moreover, functional recovery after SCI in CX_3_CR1^−/−^ mice was more rapid and sustained, implicating the importance of neuron/microglial signalling in neuronal regeneration. Therefore, in SCIs, microglia expressing the CX_3_CR1 receptor may release more substances that activate astrocytes and promote glial scar formation in favour of the alternative scenario which would be to promote functional regeneration of neuronal axons. Inhibiting CX_3_CR1 receptors at the correct temporal window post-SCI may, therefore, serve as a novel drug target to promote neuroregeneration and inhibit microglial activation.

## 14. Fractalkine and CX_3_CR1 in Alzheimer's disease

AD is a devastating progressive neurodegenerative condition that mainly affects the elderly population and is characterized by alterations in behaviour and cognitive impairment [112]. The classic hallmark of AD is extracellular plaques of misfolded amyloid-β (Aβ) protein and neurofibrillary tangles that either contribute to or are a consequence of neurodegeneration [113]. The brains of AD patients often show increased activation of microglial cells around Aβ plaques [114,115]. The natural aging process is also associated with decreased expression of neuronal FKN levels [66] which may contribute to excess microglial activation in the elderly. Neurons cultured in the absence of microglial cells show resistance to Aβ-induced neurotoxicity [116]. Fuhrmann *et al.* [117] investigated the role of CX_3_CR1 signalling in the neurotoxic effects of microglia in a triple-transgenic mouse model of AD. This mouse model displays neuronal loss in layer III of the cortex between four and six months of age. Knocking down CX_3_CR1 in these mice rescued this neuronal loss suggesting that microglia play a role in Aβ-induced neuronal death. There were a greater number of microglia around the areas of neuronal damage and this increase in microglial density preceded neuronal loss indicating that microglia are involved in the elimination process. In triple-transgenic mice that also had the CX_3_CR1 receptor knocked out, there was no increase in microglial cell densities during this period. Microglial migration velocity to the site of neuronal damage was twofold greater than in healthy areas of the cortex in both transgenic mice and in CX_3_CR1^−/−^ triple-transgenics. Once neuronal elimination was complete, however, microglial migration to the site of damage halted [117]. These observations suggest that FKN/CX_3_CR1 signalling in microglia during Aβ-induced neuronal stress is detrimental to neuronal survival.

Wu *et al.* [118] microinjected Aβ_1−40_ fibrils into the CA1 region of the hippocampus and measured increased CX_3_CR1 mRNA and protein levels, whereas FKN protein expression remained unchanged. Injecting siRNA against CX_3_CR1 along with Aβ_1−40_ fibrils not only downregulated CX_3_CR1 protein levels, but also CD11b expression. Moreover, CX_3_CR1 siRNA attenuates Aβ_1−40_-induced IL-1β release from microglia. Presumably, this IL-1β release from activated microglia contributes to the impaired LTP measured in the CA1 of rats treated with Aβ_1–40_ fibrils. Suppression of CX_3_CR1 signalling using siRNA rescues LTP expression in Aβ_1–40_-treated rats. Moreover, CX_3_CR1 siRNA also rescues spatial memory impairments in Aβ_1–40_-treated rats subjected to the Morris water maze task [118].

It is widely accepted that AD is associated with chronic inflammation which may contribute to disease aetiology and pathogenesis [119]. Targeting the CX_3_CR1 receptor early in AD may prove a fruitful drug-targeting strategy in the future. Duan *et al*. [120] assessed the levels of several chemokines at different ages in transgenic mice that develop AD-like pathology in the brain. MIP-1α levels in the hippocampus and cerebral cortex of Tg2576 mice remained the same as control at nine and 17 months of age. FKN expression, on the other hand, decreased in both brain regions while CXCL10 (IP-10) levels increased in transgenic mice. Moreover, intense CXCL10 staining co-localized with Aβ-positive plaques in the brains of transgenic animals. It is not known whether the decrease in FKN expression precedes or is a consequence of Aβ plaque formation. Several chemokines are present at high constitutive levels in the brain throughout life, and alterations in the expression of one can have downstream effects changing the levels of other chemokines. Neuronal FKN expression decreases with age [66] which may have knock-on effects on the levels of other chemokines. Therefore, preventing this natural decrease of FKN with age may represent a novel therapeutic target in the fight against AD and related dementias.

## 15. Conclusion

The FKN/CX_3_CR1 ligand/receptor pair seems to have evolved as a communication link between neurons and microglial cells. Moreover, astrocytes, in the times of inflammation, seem to upregulate FKN which also allows them to talk to microglia and neurons via CX_3_CR1 receptors. Overall, FKN appears to impart anti-inflammatory effects during neuroinflammatory events, such as stroke. This represents a novel neurotherapeutic target for such conditions. As with all cytokines and chemokines, however, it appears that the concentration and timing of administration will be crucial to maximize neuroprotection and prevent further toxicity to the system. FKN may also have an important role in normal learning and memory because it is expressed at such high levels on hippocampal neurons, in particular. High exogenous levels of FKN appear to have a dampening effect on synaptic transmission and LTP, which is activity-dependent. FKN, therefore, may function to limit over-activation of the hippocampal formation and maintain homeostasis in the neuronal network as a whole. The natural decrease of FKN in the hippocampus with age correlates with cognitive decline seen in older animals. Thus, preventing this downregulation of FKN may also represent a novel therapeutic target in the fight against AD and age-related dementias.
